# Recreational drugs and HIV in Europe: current use of recreational drugs and principal HIV guidelines related recommendations

**DOI:** 10.7448/IAS.17.4.19831

**Published:** 2014-11-02

**Authors:** Noe Garin Escriva, Cesar Velasco Muñoz, Jan Thomas De Pourcq, Belen Lopez Garcia, Josep Maria Haro Abad, Maria Antonia Mangues Bafalluy, Antoni Trilla Garcia

**Affiliations:** 1 Research and Development Unit, Parc Sanitari Sant Joan de Deu, Sant Boi, Spain; 2 Department of Preventive Medicine and Epidemiology, Hospital Clinic, Barcelona, Spain; 3 Pharmacy Department, Hospital de la Santa Creu i Sant Pau, Barcelona, Spain; 4 Pharmacy Department, Hospital del Mar – Parc de Salut Mar, Barcelona, Spain

## Abstract

**Introduction:**

Recreational drug consumption has been associated with both higher rates of risk activities related to HIV transmission and also worse adherence and management of HIV patients under HAART treatment. Moreover, relevant interactions may be present in patients under HAART treatment. Our aim is to present the European trends of drug consumption per country and age groups and assess the way drug consumption is addressed in general HIV guidelines.

**Materials and Methods:**

Last 12-month prevalence drug use was obtained from the European Monitoring Centre for Drugs and Drug Addiction for the four most consumed drugs (cannabis, cocaine, amphetamines, ecstasys). Consumption rates were collected and analyzed by country and age. Principal HIV guidelines were assessed to identify the degree of incorporation of drug use issues at three levels: transmission risk, adherence to the HAART and management of interactions. Guidelines: (a) WHO; (b) EACS; (c) BHIVA; (d) US DHHS; (e) IAS-USA; (f) GESIDA; (g) French CPG; (h) Italian CPG.

**Results:**

Data on drugs of abuse consumption was obtained from 29 European countries, with results showing relevant drug utilization in Europe. Cannabis was the most frequent drug across all countries, with 10 countries over 5% of prevalence over the last year. Other drugs prevalence accounted for about 0.5–1%, reaching up to: 2.1% for cocaine in Spain, 1.4% for ecstasy in the Netherlands and 1.1% for amphetamines in Estonia. 15–24 and 25–34 years old subgroups had the highest prevalence, although notable use of cannabis and cocaine was also found in the 35–44 and 45–54 subgroups. From the eight guidelines assessed, six considered recreational drugs at any point. Recommendations for specific drugs were given in 50% of the guidelines. From those guidelines addressing drug consumption: three assessed risk habits which related to transmission risk, six appraised issues on adherence to HAART and five comprised data on interactions between recreational drugs and HAART. Additionally, five guidelines mentioned drugs in the context of other issues, such as sexual dysfunction or HIV-associated neurocognitive impairment.

**Conclusions:**

Use of recreational drugs is frequent in Europe, not only in the younger population but also in other unexpected older subgroups. The scarce information found in the guidelines has a potential implication for patients and clinicians; therefore, there is a need to include specific recommendations about the clinical management of people living with HIV who use recreational drugs.

